# Correction: Role of inflammation in depressive and anxiety disorders, affect, and cognition: genetic and non-genetic findings in the lifelines cohort study

**DOI:** 10.1038/s41398-025-03713-9

**Published:** 2025-10-29

**Authors:** Naoise Mac Giollabhui, Chloe Slaney, Gibran Hemani, Éimear M. Foley, Peter J. van der Most, Ilja M. Nolte, Harold Snieder, George Davey Smith, Golam M. Khandaker, Catharina A. Hartman

**Affiliations:** 1 Depression Clinical & Research Program, Department of Psychiatry, Massachusetts General Hospital, Boston, USA; 2 MRC Integrative Epidemiology Unit at the University of Bristol, Bristol, UK; 3 Centre for Academic Mental Health, Bristol Medical School, University of Bristol, Bristol, UK; 4 University of Groningen, University Medical Center Groningen, Groningen, the Netherlands; 5 FRCPsych, MRC Integrative Epidemiology Unit at the University of Bristol, Bristol, UK; 6 NIHR Bristol Biomedical Research Centre, University Hospitals Bristol and Weston NHS Foundation Trust and University of Bristol, Bristol, UK; 7 Avon and Wiltshire Mental Health Partnership NHS Trust, Bristol, UK; 8 Interdisciplinary Center Psychopathology and Emotion Regulation, Department of Psychiatry, University Medical Center Groningen, University of Groningen, Groningen, the Netherlands

**Keywords:** Clinical genetics, Human behaviour

Correction to: *Translational Psychiatry* 10.1038/s41398-025-03372-w, published online 10 May 2025

The following sentence has been removed from the article:

‘However, there was slightly stronger evidence for associations between GlycAGRS and negative affect score (ps ≤ 0.015) and between CRPGRS (cis) and negative affect score (ps ≤ 0.033).’

Additionally, the ESM file has been replaced to reflect this correction.

The original article has been corrected.

## Supplementary information

Supplementary Information

